# Fluorescent *In Situ* Hybridization: A New Tool for the Direct Identification and Detection of *F. psychrophilum*

**DOI:** 10.1371/journal.pone.0049280

**Published:** 2012-11-09

**Authors:** Nicole Strepparava, Thomas Wahli, Helmut Segner, Bruno Polli, Orlando Petrini

**Affiliations:** 1 Cantonal Institute of Microbiology, Bellinzona, Switzerland; 2 Centre for Fish and Wildlife Health, University of Bern, Bern, Switzerland; 3 Cantonal Office of Hunting and Fisheries, Bellinzona, Switzerland; Cornell University, United States of America

## Abstract

*F. psychrophilum* is the causative agent of Bacterial Cold Water Disease (BCW) and Rainbow Trout Fry Syndrome (RTFS). To date, diagnosis relies mainly on direct microscopy or cultural methods. Direct microscopy is fast but not very reliable, whereas cultural methods are reliable but time-consuming and labor-intensive. So far fluorescent *in situ* hybridization (FISH) has not been used in the diagnosis of flavobacteriosis but it has the potential to rapidly and specifically detect *F. psychrophilum* in infected tissues. Outbreaks in fish farms, caused by pathogenic strains of *Flavobacterium* species, are increasingly frequent and there is a need for reliable and cost-effective techniques to rapidly diagnose flavobacterioses. This study is aimed at developing a FISH that could be used for the diagnosis of *F. psychrophilum* infections in fish. We constructed a generic probe for the genus *Flavobacterium* (“Pan-Flavo”) and two specific probes targeting *F. psychrophilum* based on 16S rRNA gene sequences. We tested their specificity and sensitivity on pure cultures of different *Flavobacterium* and other aquatic bacterial species. After assessing their sensitivity and specificity, we established their limit of detection and tested the probes on infected fresh tissues (spleen and skin) and on paraffin-embedded tissues. The results showed high sensitivity and specificity of the probes (100% and 91% for the Pan-Flavo probe and 100% and 97% for the *F. psychrophilum* probe, respectively). FISH was able to detect *F. psychrophilum* in infected fish tissues, thus the findings from this study indicate this technique is suitable as a fast and reliable method for the detection of *Flavobacterium* spp. and *F. psychrophilum*.

## Introduction

Bacteria belonging to the genus *Flavobacterium* are non-fermentative, catalase- and oxidase-positive, gram-negative bacteria that occur in abiotic and biotic compartments of many ecosystems (e.g. soil, fresh and marine water, fish). Some species, in particular *F. brevis, F. columnare, F. johnsoniae, F. branchiophilum* and *F. psychrophilum*, are ubiquitous, opportunistic pathogens that may cause disease symptoms in injured or immunologically weak animals and sometimes also in humans [1], [2], [3], [4]).

*F. psychrophilum* is a pathogenic agent causing both external and systemic infections in fish. One of the diseases caused by *F. psychrophilum* is the so-called Bacterial Cold Water (BCW) Disease, which is geographically widespread and affects a variety of fish species [5], [6]. BCW is characterized by epidermal necrosis leading to saddle-like skin lesions, usually near the dorsal fin, but also the mouth or gills may be affected, particularly in juvenile fish. The Rainbow Trout Fry Syndrome (RTFS) is a severe systemic infection that occurs in general when bacteria accumulate in the liver or spleen of salmonids. It causes high mortalities in cultured fish stocks, primarily when the infection occurs in small rainbow trout [7]. It is not yet clear, however, whether RTFS is the result of a systemic infection or an advanced form of a superficial infection.

Diagnosis of *F. psychrophilum* infection is lengthy and time-consuming, being mainly based on macroscopic and microscopic examination of fresh spleen samples and culture methods. *F. psychrophilum* is a fastidious, slow-growing, opportunistic pathogen, the growth of which is inhibited by the presence of other microorganisms; selective plates are not available and the colonies are often overgrown by other fast-growing bacteria. In addition, *F. psychrophilum* grows optimally at 15°C, an incubation temperature not routinely used in diagnostic labs [8]. As a result, *F. psychrophilum* is easily overseen during sample processing and the number of incorrect diagnoses can therefore be quite high. A rapid, sensitive and specific detection method enabling diagnosis of *F. psychrophilum* at an early stage of infection would help to prevent further spread of the disease.

Fluorescent *in situ* Hybridization (FISH) is frequently used to detect bacterial species in environmental and clinical samples and species-specific probes have been developed for the rapid identification of pathogenic species [9], [10]; FISH has already been employed to identify flavobacteria, using probes designed on 16S rRNA gene sequences targeting the *Cytophaga-Flavobacterium-Bacteroides* (CFB) group [11], [12]. So far, however, specific probes for the genus *Flavobacterium* in general or for the fish pathogenic species *F. psychrophilum* in particular are not available.

This study is aimed to develop genus- and species-specific probes that can be used to detect and identify *Flavobacterium* spp. and *F. psychrophilum* in particular, and to test the usefulness of this technique in the early diagnosis *in situ* of infections caused by *F. psychrophilum* in salmonids.

## Methods

### Strains Used

We used pure cultures of *Flavobacteria* and other bacterial species isolated from soil, water and fish, as well as clinical isolates of related and unrelated bacterial species. A list of all tested bacteria and their origin is presented in Table S1.

Water was collected in fish farms (inlets, water from fish tanks and water at the outlets of the fish farms). Swabs from immersed soil or tank surfaces were suspended in 1 ml of sterile water. For each sample 100 µl of suspension and a 1∶10 dilution thereof were plated onto CY-Agar (medium 67 DSMZ for *F. psychrophilum*: 0.3% casitone, 0.136% CaCl_2_•H_2_O, 0.1% yeast extract, 1.5% agar) as well as on Enriched Cytophaga Agar Medium (CYAM) (medium 1133 DSMZ for *F. columnare*: 0.2% tryptone, 0.05% beef extract, 0.05% yeast extract, 0.02% sodium acetate, 1.5% agar).

Fish suspected to be infected were sent by fish farmers in a container with water. A sample of their external mucus was taken and the fish were then killed by immersion in 0.01% benzocaine followed by cutting along the vertebral column allowing for the removal of the spleen. The external mucus, gills and spleens of rainbow trout (*Oncorhynchus mykiss*) and brown trout (*Salmo trutta fario* and *Salmo trutta lacustris)* were collected and homogenized separately in 200 µl of sterile water. The homogenates were plated on both CY and CYAM.

All samples were incubated at 15°C for 5 to 10 days. Growing colonies were transferred onto fresh plates and pure cultures were conserved in 1 ml skimmed milk [7% Skim Milk (Becton Dickinson, Switzerland), 10% bovine serum and 20% glycerol] at −80°C.

For FISH, symptomless fish (brown trout fario and rainbow trout) from a fish farm in Rodi (Cantonal Fish Farm, Ticino, Switzerland) in which no signs of infection were present were treated as described above. The body surfaces were swabbed using 70% ethanol to prevent contamination of the spleens by normal external bacterial flora. The spleens were removed and stored at −20°C until the time of the experiments and were then homogenized by grinding them in 200 µl of sterile water.

Approval for the animal experiments and the water collection was obtained from the Federal Veterinary Office (FVO, Switzerland) and the Ticino Cantonal Veterinary Office (Authorization 03/2010 and 04/2010).

### Identification of the Isolates

Based on growth characteristics, colonies suspected to be *Flavobacterium* spp. were transferred onto fresh plates of CY-Agar and CYAM and grown for 5 days. DNA of all samples was extracted using the Instagene kit (Bio-Rad, Hercules (CA).

Putative *Flavobacterium* strains were identified by 16S rRNA gene sequencing [13]. All other clinical and environmental isolates were identified by MALDI-TOF mass spectrometry [14], [15]. When identification by MALDI-TOF MS was not possible, identification was carried out by 16S rRNA gene sequencing.

### 16S rRNA Gene Sequencing

16S rRNA gene PCR was carried out using the universal primers uniL 26f (5′-ATTCTAGAGTTTGATCATGGCTCA-3′) and uniR 1392r (5′-ATGGTACCGTGTGACGGGCGGTGTGTA-3′) [16] PCR amplifications were carried out in a total volume of 50 µl. 25 µl of Taq PCR Master Mix (QIAGEN, Switzerland), 1.5 µl of each primer, 17 µl of water and 5 µl of DNA were mixed and the PCR was performed at the following conditions: 5 min. at 94°C, 35 cycles of 30 s at 94°C, 30s at 52°C and 1 min. at 72°C with a final elongation of 7 min. at 72°C. Purification of PCR products was carried out with PCR clean-up NucleoSpin® ExtractII (Macherey-Nagel, Germany). Sequencing was performed using the BigDye Terminator v1.1 Cycle Sequencing kit (Applied Biosystems, Switzerland) according to the manufacturer’s instructions. Reactions were carried out in a total volume of 15 µl containing 3 µl of BigDye®, 1.5 µl of BigDye® Buffer, and 2.4 µl of a 1 µM primer solution. The same primers used for PCR were also used for the 16S rRNA gene sequencing. Thermal cycling conditions were 1 min at 96°C, followed by 25 cycles of 10s at 96°C, 5s at 50°C and 4 min at 60°C. The sequencing products were purified on a 0.025 mm membrane filter in a Tris-EDTA buffer solution (pH 8) before sequencing with Hi-Di™ Formamide (Applied Biosystems) on a AB Prism 310 Genetic Analyzer (Applied Biosystems). The obtained sequences were compared with data included in GenBank (http://blast.ncbi.nlm.nih.gov/).

### Probes Used

Oligonucleotide FISH probes were manually designed by aligning all 16S rDNA of *Flavobacterium* strains of interest. Sequences were downloaded from GenBank for the following species: *Flavobacterium psychrophilum* (AY662493, AB297676, AB297673), *F. branchiophilum* (D14017), *F. columnare* (AM230485, AB015481, AB010951, AB180738), *F. granuli* (AB180738), *F. johnsoniae* (AM921621), *F. degerlachei* (AJ441005), *F. flevense* (AJ440988), *F. frigidarium* (EU000241), *F. frigoris* (AJ440988), *F. hibernum* (L39067), *F. hydatis* (M58764), *F. limicola* (AB075230), *F. pectinovorum* (AM230490), *F. succinicans* (AM230492), and *F. omnivorum* (AF433174). Sequence alignment was performed using MEGA4. Probes were named by their position after alignment of all *Flavobacterium* sequences using *Escherichia coli* HM371196 as the outgroup.

The possible target regions were chosen by evaluating which region within the 16S rRNA secondary structure of *E. coli* would be most suitable for probe design [17]. This led to the construction of the generic *Flavobacterium* probe (“Pan-Flavo”: Flavo285; Table 1). Pan-Flavo was labeled with Cyanine dye (CY3) at the 5′ end.

**Table 1:** Probes used, target microorganisms and DNA target regions. [Cyanine dye (CY3); Carboxyfluorescein (FAM)].

Name	Target microorganism	Target region in E.coli*	Length	Sequence	Labeling
Flavo285	*Flavobacterium* spp.	230	17	5′-GACCCCTACCCATCRTH-3′	CY3
FlavoP77	*F. psychrophilum*	138	22	5′-AGTGTGTTGATGCCAACTCACT-3′	FAM
FlavoP477	*F. psychrophilum*	532	19	5′-ACTTATCTGGCCGCCTACG-3′	FAM

* *E.coli*, GenBank sequence HM371196.

Two probes (FlavoP77 and FlavoP477, Table 1) were designed for the specific detection of *F. psychrophilum* using the same 16S rDNA alignment of sequences as described above, using, however, seven additional *F. psychrophilum* strains (AB297675, AB297484, AB297483, AB297674, AB297671, AB297672, AB297494) to cater for internal *F. psychrophilum* variability. These two oligonucleotide probes were then labeled with Carboxyfluorescein (FAM) at the 5′ end.

To test for a possible cumulative effect of different fluorochromes applied on the same slide, a Pan-Flavo probe was constructed with the same primer as above, but without labeling.

The sensitivity and specificity of the Pan-Flavo and *F. psychrophilum* probes were tested on several *Flavobacterium* species (*F. psychrophilum, F. columnare, F. branchiophilum, F. johnsoniae, F. fryxellicola, F. frigidimaris, F. aquatile, F. psychrolimnae, F. succinicans, F. aquidurense, F. hercynium, F. hydatis, F. limicola, F. pectinovorum) and Chryseobacterium* spp. strains isolated from our samples as well as on non-*Flavobacteriaceae* isolates (Table S1).

The specificity of the probes was also tested *in silico* using the Ribosomal Database Project (RDP) [18], [19], thus providing evidence that the designed probes match the sequences present in the database and therefore making them suitable for the *in vivo* assays.

The probes were synthesized by Microsynth (Balgach, Switzerland).

### FISH Conditions

Each putative *Flavobacterium* colony was resuspended in 200 µl of sterile water. Ten microliters were added in a well of ten-well immunofluorescence microscopy slides (bioMérieux, Geneva, Switzerland), air-dried and dehydrated sequentially in 50%, 70%, and 96% ethanol during 3 min for each condition. To determine stringent hybridization conditions, a formamide series was carried out with a pure culture of *F. psychrophilum*. The best results were obtained at a formamide concentration of 30%. 10 µl hybridization solution (0.9 M sodium chloride, 20 mM Tris/HCl pH 7, 30% formamide, water, 0.01% SDS) containing 50 ng of the oligonucleotide probe were added to each well and the sample incubated for 12 to 16 hours in an isotonically equilibrated, humid Falcon tube (Greiner bio-one, Verridia, Switzerland) at 46°C. After the incubation step, the slides were kept at 48°C for 20 min in 50 ml of washing solution (150 mM sodium chloride, 100 mM Tris/HCl pH 7, 5 mM EDTA pH 8, 0.01% SDS, water up to 50 ml), rinsed with distilled water, air-dried, and stained with an aqueous solution of 4′-6-diamidino-2-phenylindole (DAPI) (Fluka, Switzerland) for 7 min (10 µl ml^−1^); after DAPI staining, slides were rinsed again with distilled water, air dried and mounted with Citifluor (Citifluor Ltd., London, UK). Slides were screened for fluorescence using an Axiolab microscope (ZEISS, Switzerland) equipped with filters for FITC (excitation 494 nm; emission 518 nm), Cy3 (excitation 562 nm; emission 576 nm) and DAPI (excitation 360 nm; emission 456 nm). *Flavobacterium psychrophilum* (DSM 3660), environmental samples of *Flavobacterium* spp. and *Chryseobacterium* spp. were used as controls.

### Quantification of Bacteria

Optical Density (OD_595_) of pure *F. psychrophilum* bacterial suspension (n = 10) was adjusted at 0.3 (±0.02) with a Perkin Elmer spectrophotometer (Perkin Elmer UV/VIS Spectrometer Lambda 2S, Waltham, MA). DNA was extracted from 1 ml of suspension with the QIAGEN tissue and blood kit (QIAGEN). The total amount of DNA was quantified with a Nanodrop spectrophotometer (ND1000, Witec, Switzerland) and divided by 3.137×10^−6 ^ng [the weight of one *F. psychrophilum* genome (genome size 2′861’988 bp [20])]. This yields the number of bacteria present in one ml of the starting OD suspension. Thus, an OD of 0.3 corresponds to 3×10^9^±7×10^8^ cells.ml^−1^ [21].

To determine the limit of detection and evaluate the goodness of FISH for diagnostic purposes, we plated out aliquots of the bacterial suspensions and assessed their growth, as cultures are currently used in veterinary laboratories to assess the presence of the pathogen in fish samples.

### Limits of Detection for Suspensions from Pure Cultures

Pure cultures of *F. psychrophilum* grown on CYAM agar were adjusted in sterile water at 3×10^9^ cells.ml^−1^ (OD 0.3). Twenty serial two-fold dilutions were prepared and 100 µl of each were plated on CYAM; 10 µl of each dilution were put on a ten-well immunofluorescence microscopy slide. *F. psychrophilum* (DSM3660) and water were used as positive and negative controls, respectively.

### Limits of Detection in Fish Tissues

Serial dilutions of a stock suspension of *F. psychrophilum* (9.4×10^7^ cells.ml^−1^) were used for the experiment. For each serial dilution one spleen and one *F. psychrophilum* isolate were used. In a 200 µl Eppendorf, 10 µl of ground spleen were seeded with 10 µl of a bacterial suspension: 10 µl of the final suspension were placed on a ten-well immunofluorescence microscopy slide and 10 µl were plated on CYAM medium. A 1∶32 dilution of the stock suspension was used as a positive control and a mix of 10 µl water and 10 µl of spleen was used as a negative control.

### Diagnosis of Putative Infections by FISH

During 2011–2012, fish samples from Swiss fish farms were collected periodically to check for the systemic infection by *F. psychrophilum*. In addition, each potential infection reported by fish farmers was screened by FISH with the Pan-Flavo and *F. psychrophilum* probes to check for the presence of the pathogen on skin and spleen tissues.

The entire spleen and a sample of skin mucus were homogenized individually in 200 µl of sterilized water. 10 µl of each homogenate were added to a ten-well immunofluorescence microscopy slide and 100 µl were plated on CYAM agar medium for control.

### Detection of *Flavobacterium* and *F. psychrophilum* in Paraffin Embedded Tissues

Serial sections of paraffin-embedded tissues from diseased fish were prepared and one section was stained with Giemsa. Pretreatment of FISH staining followed the protocol of Ridderstrale et al. [22]. Briefly, slides were heated at 65°C for 1 hour, immersed in 0.2 M HCl for 15 min, rinsed with water, incubated in 0.01 citrate buffer (pH 6.0) at 100°C for 90 s and at 90°C for 7 min. Slides were then immersed in 70% and 100% ethanol at 4°C for 3 min each, washed in standard saline citrate (SSC, 2X) and incubated in 0.5 mg/ml pepsin (Merck, Switzerland) in NaCl 0.9% (pH 2) for 20 min at 37°C. At the end, samples were dehydrated in 70% and 100% ethanol during 2 min each. FISH was carried out using the same method described for pure cultures.

### Statistical Methods

Sensitivity (SE), specificity (SP), positive predictive values (PPV) and negative predictive values (NPV) for all probes were calculated using DAG-Stat.xls [23], [24], [25]. Alignments and phylogenetic tree construction were carried out using MEGA version 4 [26]. The limit of detection for pure culture and for spiked spleen was defined as the fifth percentile of all analyzed positive and negative samples. Receiver operating characteristic (ROC) analysis was done using SPSS version 17.0 (SPSS Inc., Chicago, IL, USA).

## Results

### Simultaneous Use of *F. psychrophilum* (FlavoP77 and FlavoP477) and Pan-Flavo (Flavo285) Probes

Two conserved regions within the *F. psychrophilum* 16S rDNA, with species-specific sequences, were chosen and tested individually. Results from a first experiment carried out on 10 *F. psychrophilum* strains in duplicates for each probe were not reproducible due to a too low fluorescence and an immediate loss of signal: therefore, in a second step a combination of both *F. psychrophilum* probes as well as a combination of the two *F. psychrophilum* probes with the Pan-Flavo probe were tested. Stable and accurate results were obtained using the two *F. psychrophilum* probes; the addition of the Pan-Flavo probe was, however, crucial to obtain the optimal fluorescence at which these bacteria can be easily seen through the microscope. To test whether or not the improved staining results, with the combination of the three probes, is due to a potentially cumulative fluorescence caused by the simultaneous presence of two types of fluorochromes (CY3 and FAM), we prepared a helper oligonucleotide probe with the same sequence as the Pan-Flavo probe but without a fluorescent label: this led to the same results as with the two fluorochromes. Further tests were carried out with the three probes available using them simultaneously, with essentially the same outcome.

**Figure 1:**
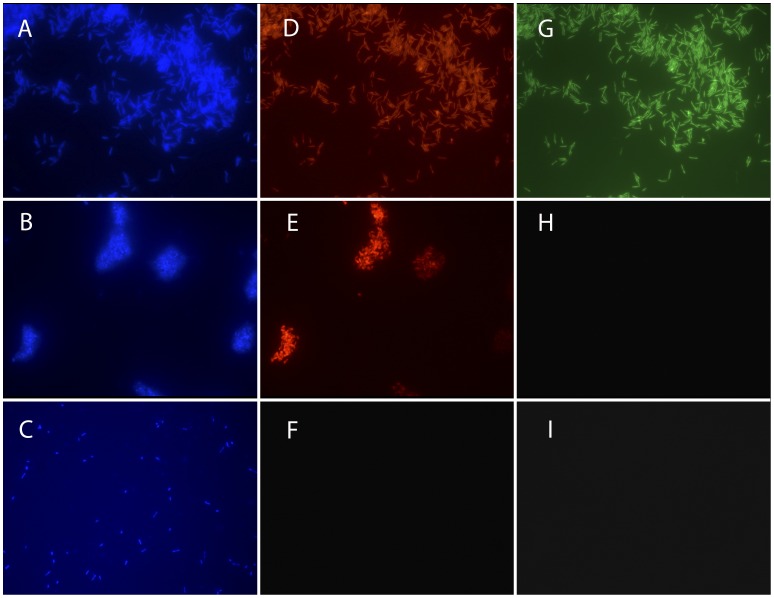
FISH assays of pure cultures. DAPI staining (A, B, C); Pan-Flavo probe (D, E, F); *F. psychrophilum* probes (G, H, I) (100x). *F. psychrophilum* (DSM3660) (A, D, G); *Flavobacterium* spp. (B, E, H); *Chryseobacterium* spp. (C, F, I).

Tests performed on 352 isolates (50 strains of *F. psychrophilum*, 226 *Flavobacterium* spp. and 76 other bacterial species) demonstrated that the Pan-Flavo and *F. psychrophilum* probes were highly sensitive and specific (98% and 100% for Pan-Flavo probe and 100% and 98% for *F. psychrophilum* probes) (Table 2). PPV and NPV values were 100% and 95%, respectively, for the Pan-Flavo probe and 91% and 100% for the specific *F. psychrophilum* probes. The probes showed no recognizable cross-reactions with other bacterial species (Figure 1).

**Table 2:** Agreement between FISH and 16S rDNA sequencing (SEQ, used as gold standard) in the experiments carried out with the Pan-Flavo (Flavo285) probe and the combination of two *F. psychrophilum* (FlavoP77, FlavoP477) probes.

Pan-Flavo	FISH +	FISH −	Total	
SEQ +	272	4	276	SE: 98%
SEQ −	0	76	76	SP: 100%
Total	272	80	352	
	PPV: 100%	NPV: 98%		
FlavoP77+ FlavoP477	FISH +	FISH −	Total	
SEQ +	50	0	50	SE: 100%
SEQ −	5	297	302	SP: 98%
Total	55	297	352	
	PPV: 91%	NPV: 100%		

SE: sensitivity; SP: specificity; PPV: positive predictive value, NPV: negative predictive value.

Only 4 out of 276 *Flavobacterium* sp. strains did not react with the Pan-Flavo probe. Each strain was tested twice: the first essay was negative and the second could not unequivocally identify the strains as *Flavobacterium* sp. No mismatches were present in the alignment of the probe with the target sequence, therefore we have no clear explanation for this result: the error is approximately 1% (4 wrong identifications over 352 total strains) and, in our opinion, may be ascribed to natural variations among the samples studied. 5 out of the 352 strains tested were erroneously identified as *F. psychrophilum* but 16 s rRNA gene sequencing showed that they belonged to *Flavobacterium* sp. other than *F. psychrophilum*.

### Limits of Detection for Pure Culture Suspensions

**Figure 2:**
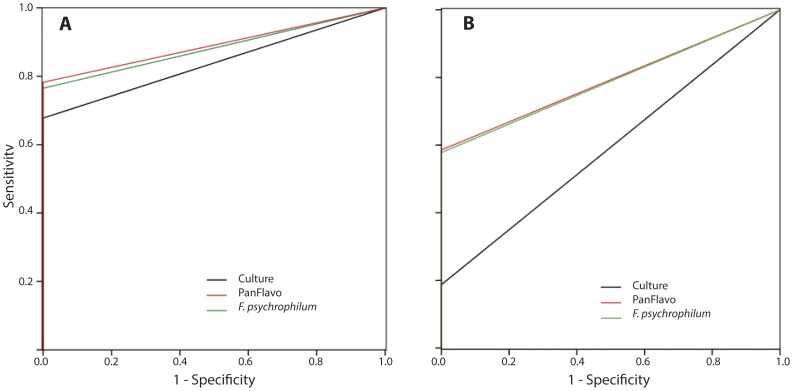
ROC curves for cell suspension of pure strains; area under the curve (AUC) for FISH: 0.89, for culture method: 0.79. (A). ROC curves for spiked spleens; AUC for FISH: 0.84, for culture method: 0.6 (B).

The LOD was established by investigating serial two-fold dilutions. In 95% of the tested cases (15 strains in duplicate), the LOD for the Pan-Flavo and *F. psychrophilum* probes was 7.3×10^5^ cells.ml^−1^ by FISH; the LOD of the cultural method was only 3×10^9^ cells*ml^−1^ (93% of the tested strains). ROC analysis (Figure 2A) showed a statistically significant higher sensitivity of the FISH method compared to culture (areas under the curve (AUC) for the Pan-Flavo probe: 0.89; for the *F. psychrophilum* probe: 0.88; culture method: 0.84).

### Limit of Detection in Fish Tissues

The LOD for the Pan-Flavo and *F. psychrophilum* probes applied to fish tissue samples was 2.9×10^6^ cells.ml^−1^. An even lower LOD, 1.5×10^6^ cells.ml^−1^ was reached with the PanFlavo probe in 80%, and with the *F. psychrophilum* probes in 70% of the cases. LOD was 3×10^9^ cells.ml^−1^, with only 40% of positive cultures. According to the ROC analysis, FISH appears to be more sensitive than culture, both for the Pan-Flavo and *F. psychrophilum* probes (Figure 2B). Likewise, the AUC values of both the Pan-Flavo and the *F. psychrophilum* probes were higher than those of the culture method (0.79 for FISH vs. 0.59 for culture).

### Diagnosis of the Disease

**Figure 3:**
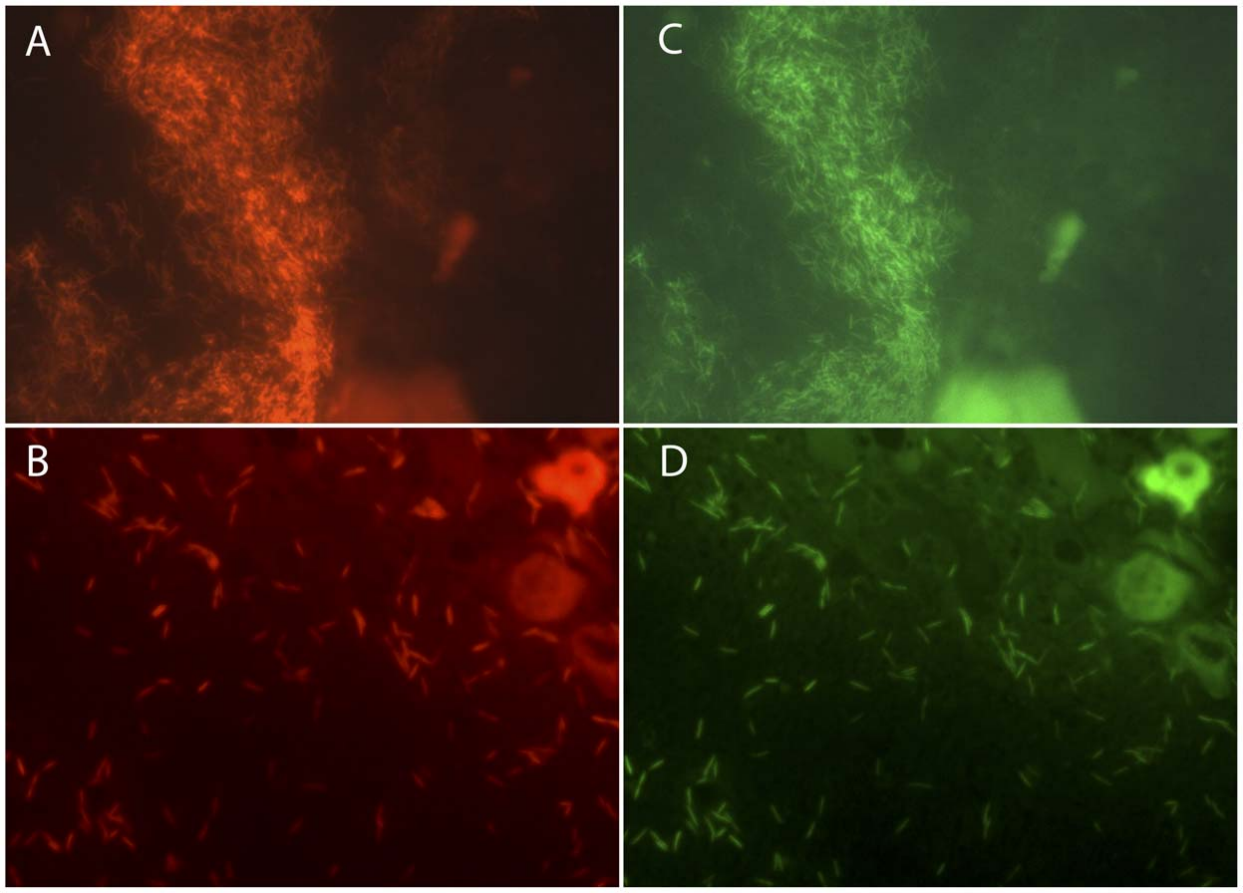
FISH assay on infected fish tissues. Pan-Flavo probe (A, B); *F. psychrophilum* probes (C, D). *F. psychrophilum* on skin (A, C) and *F. psychrophilum* in a spleen (B, D).

FISH was very successful in detecting and identifying *F. psychrophilum* from fresh samples. Diagnosis by FISH was available within 24 hours as compared to 4 to 10 days with the culture method. The *F. psychrophilum* probes detected the pathogen in 13 cases of BCW and RTFS (Figure 3). In 9 cases, the FISH-based diagnosis was confirmed by culture, while in 1 case no growth in culture was seen. The remaining 3 cases were repeated samplings from the same fish farm: confirmation of the infection was possible with culture only after a fourth sampling more than one month after diagnosis by FISH.

### Detection of *Flavobacterium* and *F. psychrophilum* from Paraffin Embedded Tissues

Five (4 positive and 1 negative) samples were fixed in paraffin, and 3–4 sections of each bloc were cut and mounted on a slide. Out of 11 samples, 7 were correctly detected as positive, 2 were correctly detected as negative and 2 samples were false negatives.

## Discussion

The probes designed in this study, specifically targeting the genus *Flavobacterium* and the pathogenic species *F. psychrophilum*, are highly sensitive and specific (98% and 100% for Pan-Flavo, 100% and 98% for the *F. psychrophilum* probes) and allow correct identification of *Flavobacterium* spp. and *F. psychrophilum* in culture. The same probes were also used successfully to screen fish tissues for the presence of *Flavobacterium* sp. or *F. psychrophilum*. Compared to currently used diagnostic methods, FISH was rapid, as the results were obtained within 24 hours, as compared with 5 to 10 days needed to culture the bacteria. Thus the application of FISH offers a valuable tool for the rapid detection of *Flavobacterium* spp. and in particular *F. psychrophilum* in fish tissue.

Combining the two *F. psychrophilum* probes with the Pan-Flavo probe was crucial for a reliable detection of *F. psychrophilum*. The need for multiple labeling to increase signal strength has already been described by other authors [27]. It is assumed that the second probe enhances the annealing of the diagnostic probe with its corresponding rRNA [28]. Generally, a “helper” probe targets the sequence of a region directly adjacent to the diagnostic probe site [29]: however, the Pan-Flavo probe which, in our case, acted as a helper, is almost equidistant to both *F. psychrophilum* specific probes. This was not expected and we hypothesize that the effect may be related to the tertiary structure of the target region.

No cross-reaction was observed between the Pan-Flavo probe or the *F. psychrophilum* probes with other taxonomically closely related species that might be present in environmental and clinical samples. In our evaluation of sensitivity and specificity, we deliberately chose species known to be part of the aquatic environmental microbiota and we did not test opportunistic human and animal pathogens closely related with *Flavobacterium* such as *Capnocytophaga*, found in the mammal oropharyngeal tract [30], or marine environment organisms such as *Tenacibaculum* [31]. Because of the particular ecological niche occupied by these species we do not expect them to be present in fish samples.

The minimal concentration of *F. psychrophilum* cells needed in a sample to yield a positive result by FISH is lower than for the culture method (7.3×10^5^ cells ml^−1^ vs. 3×10^9^ cells ml^−1^ for water, and 2.9×10^6^ vs. 3×10^9^ cells ml^−1^ for spleens). ROC analysis confirmed that FISH is more sensitive than culture (AUC for FISH 0.89 vs. 0.84 for culture with suspension of pure cultures and 0.79 for FISH vs. 0.59 for culture with spiked spleens). FISH also yielded reproducible results within and between isolates. This is not the case for the culture method, which showed variability even for one and the same isolate, with growth not always being reproducible.

In medical microbiology, FISH is frequently used as a cheap, easy and rapid method to identify pathogens directly in blood cultures; in these settings LODs are quite low, being approximately 1000 microorganism per ml ([32]). In our study, we detected the bacteria in spleen homogenates, a more difficult diagnostic matrix than blood. Indeed, for all three probes, the LOD for spiked spleens was higher than for pure culture suspensions. The LOD in spleens was 2.9*10^6^ cells/ml, mostly because of a rather high background fluorescence probably caused by the presence of muscular tissue and collagen. This is in agreement with a study by Marquardt and Wold [33] who used Raman spectroscopy to quantify collagen, fat and pigments such as carotenoids that were reportedly highly autofluorescent.

FISH detected *F. psychrophilum* within 24 hours in all infected samples: fresh samples of spleen and mucus are particularly well suited for analysis. The rapid diagnosis by FISH allows starting a timely and adequate treatment of the infection and could thus lead to better results in fish survival. FISH shows also a great potential for use on fixed tissues in retrospective studies of infections by *Flavobacterium*. Results, however, may be difficult to interpret due to the high background fluorescence of tissues. We had only four confirmed cases of *F. psychrophilum* infection available to test the method. In three of these cases the pathogen could be detected in spleen and liver tissues; while in one case the detection of *F. psychrophilum* was not possible. This could be explained by an inhomogeneous distribution of the infection in the tissues studied or by a bacterial count below LOD. On the other hand, the high background fluorescence could lead to false negative results when screening tissue sections.

FISH is an easy, fast and non-labour intensive technique. It does not require particular technical skills and is already used in many different fields such as clinical, veterinary, food and environmental microbiology [10], [32], [34], [35], [36]. Here we describe for the first time the successful use of FISH probes for the detection of *Flavobacterium* spp. and *F. psychrophilum* in environmental and tissue samples. The method described allows a fast and reliable qualitative detection of *Flavobacterium* spp. and *F. psychrophilum* in potentially infected tissues. While the method is particularly convenient in the diagnostic field, it does not replace culture, which is still needed for antibiotic sensitivity testing and other physiological studies.

## Supporting Information

Table S1 **Species investigated, number (N) and origin of strains (352 isolates in total).**
(DOC)
