# Supplementary material for: Fluorescent In Situ Hybridization: A New Tool for the Direct Identification and Detection of F. psychrophilum
Source: PLoS One. 2012 Nov 9;7(11):e49280. doi: 10.1371/journal.pone.0049280 (PMC3494677; doi:10.1371/journal.pone.0049280)
Supplement: Table S1 — Species investigated, number (N) and origin of strains (352 isolates in total). (DOC) [file pone.0049280.s001.doc]

Table S1. Species investigated, number (N) and origin of strains (352 isolates in total).

| **Species** | **N** | **Origin** |
| --- | --- | --- |
| *Acidovirax* sp. | 1 | Swiss fish farms |
| *Aeromonas bestiarum* | 1 | Swiss fish farms |
| *Aeromonas eucrenophila* | 1 | Swiss fish farms |
| *Aeromonas media* | 1 | ATCC 33907 |
| *Aeromonas schuberti* | 1 | ATCC 43700 |
| *Aeromonas veronii* | 1 | ATCC 35624 |
| *Aeromonas hydrophila* | 1 | ATCC 7966 |
| *Aeromonas caviae* | 1 | ATCC 15468 |
| *Aeromonas sobria* | 1 | CIP 7433 |
| *Aeromonas salmonicida* | 1 | ATCC 33658 |
| *Aeromonas jandaei* | 1 | ATCC 49568 |
| *Aeromonas trota* | 1 | ATCC 49657 |
| *Aeromonas allosaccharophila* | 1 | DSM 11576 |
| *Aeromonas encheleia* | 1 | DSM 11577 |
| *Aeromonas culicicola* | 1 | CIP 107763 T |
| *Aeromonas simiae* | 1 | CIP 107798 T |
| *Aeromonas molluscorum* | 1 | DSM 17090 |
| *Aeromonas bivalvum* | 1 | Cantonal Institute of Microbiology |
| *Aeromonas popoffi* | 1 | LMG 17541 T |
| *Aeromonas* spp. | 3 | Swiss fish farms |
| *Burkholderia* sp. | 1 | Human infection |
| *Chryseobacterium* spp. | 11 | Swiss fish farms |
| *Citrobacter freundii* | 1 | Human infection |
| *Enterococcus* sp. | 1 | Human infection |
| *Enterococcus faecium* | 2 | Human infection |
| *Enterobacter cloacae* | 1 | Human infection |
| *Escherichia coli* | 4 | Human infection |
| *Flavobacterium aquidurense* | 1 | DSM 18293^T^ |
| *Flavobacterium columnare* | 2 | (Institut National de la Recherche Agronomique and College of Veterinary Medicine Mississippi State) |
| *Flavobacterium branchiophilum* | 1 | FL-15 (Institut National de la Recherche Agronomique) |
| *Flavobacterium frigidimaris* | 1 | KUC-1 (Institut National de la Recherche Agronomique) |
| *Flavobacterium hercynium* | 1 | DSM 18292^T^ |
| *Flavobacterium hydatis* | 1 | DSM 2063^T^ |
| *Flavobacterium johnsoniae* | 1 | DSM 20694 (Institut National de la Recherche Agronomique) |
| *Flavobacterium limicola* | 1 | DSM 15094^T^ |
| *Flavobacterium pectinovorum* | 1 | DSM 6368^T^ |
| *Flavobacterium psychrolimnae* | 1 | CIP 108326 (Institut National de la Recherche Agronomique) |
| *Flavobacterium psychrophilum* | 50 | Swiss fish farms, DSM3660^T^, Laboratory of Aquatic Pathobiology |
| *Flavobacterium succinicans* | 1 | DSM 4002^T^ |
| *Flavobacterium* spp. | 215 | Swiss fish farms |
| *Janthinobacterium* sp. | 1 | Swiss fish farms |
| *Klebsiella pneumophila* | 3 | Human infection |
| *Massilia* spp. | 2 | Swiss fish farms |
| *Pasteurella multocida* | 1 | Human infection |
| *Pseudomonas aeruginosa* | 3 | Human infection |
| *Salmonella* spp. | 6 | Human infection |
| *Serratia marcescens* | 2 | Human infection |
| *Shigella* spp. | 2 | Human infection |
| *Sphingomonas* sp. | 1 | Swiss fish farms |
| *Staphylococcus epidermidis* | 1 | Human infection |
| *Staphylococcus hominis* | 1 | Human infection |
| *Staphylococcus aureus* | 3 | Human infection |
| *Staphylococcus sp. (coagulase negative)* | 2 | Human infection |
| *Streptococcus mitis* | 1 | Human infection |
| *Streptococcus oralis* | 1 | Human infection |
| Other (uncultured bacterium) | 2 | Swiss fish farms |
